# Construction of Red Fox Chromosomal Fragments from the Short-Read Genome Assembly

**DOI:** 10.3390/genes9060308

**Published:** 2018-06-20

**Authors:** Halie M. Rando, Marta Farré, Michael P. Robson, Naomi B. Won, Jennifer L. Johnson, Ronak Buch, Estelle R. Bastounes, Xueyan Xiang, Shaohong Feng, Shiping Liu, Zijun Xiong, Jaebum Kim, Guojie Zhang, Lyudmila N. Trut, Denis M. Larkin, Anna V. Kukekova

**Affiliations:** 1 Illinois Informatics Institute, University of Illinois at Urbana-Champaign, Urbana, IL 61801, USA; rando2@illinois.edu; 2 Department of Animal Science, College of Agricultural, Consumer and Environmental Sciences, University of Illinois at Urbana-Champaign, Urbana, IL 61801, USA; krnommers@gmail.com (N.B.W.); jjohnso@illinois.edu (J.L.J.); bastoun2@illinois.edu (E.R.B.); 3 Department of Comparative Biomedical Science, Royal Veterinary College, London NW1 0TU, UK; mfbelmonte@rvc.ac.uk (M.F.); dlarkin@rvc.ac.uk (D.M.L.); 4 Department of Computer Science, College of Engineering, University of Illinois at Urbana-Champaign, Urbana, IL 61801, USA; mprobson@illinois.edu (M.P.R.); rabuch2@illinois.edu (R.B.); 5 China National Genebank, BGI -Shenzhen, Shenzhen 518083, Guangdong, China; x.xiang@uq.edu.au (X.X.); fengshaohong@genomics.cn (S.F.); liushiping@genomics.cn (S.L.); xiongzijun@genomics.cn (Z.X.); guojie.zhang@bio.ku.dk (G.Z.); 6 Department of Stem Cell and Regenerative Biology, Konkuk University, Seoul 05029, Korea; jbkim@konkuk.ac.kr; 7 Section for Ecology and Evolution, Department of Biology, Universitetsparken 15, University of Copenhagen, DK-2100 Copenhagen, Denmark; 8 State Key Laboratory of Genetic Resources and Evolution, Kunming Institute of Zoology, Chinese Academy of Sciences, Kunming 650223, China; 9 Institute of Cytology and Genetics of the Russian Academy of Sciences, Novosibirsk 630090, Russia; trut@bionet.nsc.ru

**Keywords:** *Vulpes vulpes*, comparative genomics, chromosome assembly, 10K Genomes Project, evolutionary breakpoints, Carnivora, Canidae, chromosome evolution, genome assembly

## Abstract

The genome of a red fox (*Vulpes vulpes*) was recently sequenced and assembled using next-generation sequencing (NGS). The assembly is of high quality, with 94X coverage and a scaffold N50 of 11.8 Mbp, but is split into 676,878 scaffolds, some of which are likely to contain assembly errors. Fragmentation and misassembly hinder accurate gene prediction and downstream analysis such as the identification of loci under selection. Therefore, assembly of the genome into chromosome-scale fragments was an important step towards developing this genomic model. Scaffolds from the assembly were aligned to the dog reference genome and compared to the alignment of an outgroup genome (cat) against the dog to identify syntenic sequences among species. The program Reference-Assisted Chromosome Assembly (RACA) then integrated the comparative alignment with the mapping of the raw sequencing reads generated during assembly against the fox scaffolds. The 128 sequence fragments RACA assembled were compared to the fox meiotic linkage map to guide the construction of 40 chromosomal fragments. This computational approach to assembly was facilitated by prior research in comparative mammalian genomics, and the continued improvement of the red fox genome can in turn offer insight into canid and carnivore chromosome evolution. This assembly is also necessary for advancing genetic research in foxes and other canids.

## 1. Introduction

At the turn of the millennium, the potential for mammalian comparative genomics to offer significant insights into both basic (e.g., adaptation and species formation) and applied (e.g., biomedical and agricultural) biology was already apparent [1]. However, early genome sequencing projects were costly investments, and mammalian genome sequencing projects were especially demanding given mammalian genomes’ size and complexity. Thus, the original mammalian genome sequencing projects targeted popular model species such as human [2,3] and mouse [4]. Initial interest in comparative mammalian genomics was catalyzed by the recognized potential for comparative analysis to reveal regions of evolutionary constraint and to support the annotation of the human genome, but the high costs associated with genome assembly using Sanger sequencing technology limited the original comparative assembly project to the assembly of genomes from only 29 eutherian mammals [5]. More recently, the advent of low-cost, high-throughput next-generation sequencing (NGS) technology has revolutionized the scale at which comparative genomic projects can be approached. The effect of NGS technology on the scope of mammalian genomics is particularly manifest in the 10K Genomes Project [6], which seeks to sequence and assemble 10,000 vertebrate genomes and to ensure that every vertebrate genus is represented. As of 2015, 111 mammalian genome assembly projects, of which 105 were placental mammals, had been completed or were underway [7]. With this shift, many species outside the axis of traditionally-studied models have become the targets of mammalian genome sequencing projects. 

One such species selected for genome sequencing through the Genome 10K Project is the red fox (*Vulpes vulpes*). In addition to being the widest-distributed wild terrestrial carnivore [8,9], the red fox has been bred to live on farms [10] and was also the subject of a unique experimental breeding program that began in 1959 at the Institute of Cytology and Genetics in Novosibirsk, Russia. For over 50 generations, one fox population has been bred to exhibit tame behavior towards humans similar to that of dogs while another has been bred to show heightened aggressive behavior towards humans [11]. Adaptation and response to selection in the red fox is therefore of interest not only to wildlife ecology, but also fields including behavioral and evolutionary genetics. The draft red fox genome assembly, which is currently in version 2.2 and known as vv2.2, has already proven valuable for studies of fox behavioral genetics [12], immunological adaptation [13], and population diversity [14]. 

While NGS can be credited with the proliferation of mammalian genome assemblies, the shift from Sanger to NGS technology is not without trade-off. Genome sequencing projects utilizing Sanger technology generated long sequencing reads (up to 800 bp), and also often included the development of physical or linkage maps. The length and low error rate [15] of Sanger reads rendered the de novo assembly of large fragments a computationally tractable problem with the overlap-layout-consensus (OLC) algorithm, which identifies a single best path through the sequencing reads [16]. This algorithm was used in the assembly of early eutherian mammal genome projects: for example, the dog genome [17] was assembled with ARACHNE [18,19], and the cat genome [20] with ARACHNE and Phusion [21]. By comparison, genomes today are often sequenced with short-read, error-prone technology whose limitations are offset by the reduced cost of sequencing at a very high coverage [15,22]. The OLC algorithm, however, becomes intractable if applied to the short-read data generated in modern genome sequencing projects because the short reads cause a proliferation of possible paths in repetitive regions [16,23]. Instead, short-read NGS genome assemblers rely on a heuristic approach to assembly that includes the deconstruction of the reads into *k*-mers followed by the traversal of a de Bruijn graph where the *k*-mers are the nodes [16,23,24]. This algorithm loses information by deconstructing the reads and is more susceptible to the introduction of errors by heterozygosity and repeats than the OLC approach is [25]. Utilizing libraries with a range of insert sizes, including long-range mate-pair libraries, is one critical step for reducing the uncertainty arising from the use of short reads to improve assemblies and allow for assembly across repetitive regions, but it does not solve this problem [22,26]. Thus, the shift from Sanger sequencing to short-read NGS technologies has precipitated a new set of bioinformatic challenges related to assembly that affect the content of de novo assemblies in predictable ways.

The red fox genome was sequenced with short-read Illumina technology using 15 libraries with insert sizes ranging from 170 to 20,000 bp. Assembly was conducted using SOAPdenovo2, which uses de Bruijn graphs [27]. The genome assembly reports high coverage of 94X and a long scaffold N50 of 11.80 Mbp, but it is fragmented into 676,878 scaffolds [12]. The fragmentation of de novo assemblies sequenced with short reads typically occurs in repetitive regions [22,24] and is expected to introduce downstream challenges, such as the prediction of genes whose exons are split across multiple scaffolds [28]. Additionally, in the red fox genome, at least 70 of the 500 largest scaffolds (approximately 50 Kbp or larger) are chimeric [12], meaning they are likely to contain sequence from multiple fox chromosomes. Chimerism represents another problem known to affect de novo assemblies sequenced with short reads [22,27], as do within-chromosome structural errors such as the introduction of spurious rearrangements or segmental duplications [29,30,31]. Thus, although the high quality of the red fox genome assembly demonstrates once again the value of high-throughput, short-read technologies to mammalian genomics, the challenges of assembly from short reads have introduced some limitations that must be addressed in order to develop this genomic resource to its full potential.

The preferred outcome of improving the assembly would be to develop a full chromosomal assembly for the red fox. In the past, the large fragments assembled with Sanger sequencing facilitated the construction of chromosomes. The shift to short-read NGS technology has positioned the assembly of small fragments into chromosomes as a significant challenge in bioinformatics [32]. However, just as the assembly of additional genomes adds probative power to comparative genetic studies, comparison to assembled genomes can facilitate the assembly of chromosomes, or at least of chromosome-scale fragments. The program Reference-Assisted Chromosome Assembly (RACA), for example, utilizes synteny between the target genome of interest, a reference, and an outgroup to identify fragments that may be conserved. It then combines this syntenic information with the mapping of sequencing libraries made with a variety of insert sizes onto the genome of interest to identify the regions of the scaffolds that are robustly supported by both synteny and sequencing. In this way, the RACA pipeline can resolve assembly errors by breaking scaffolds into ungapped fragments colinear between the reference and target species (conserved blocks) from which it constructs larger, gapped colinear sequences (syntenic fragments) from the sequence content of multiple scaffolds. RACA then merges its syntenic fragments to produce sequences corresponding to predicted chromosome fragments (RACA fragments). RACA has previously been used in the development of chromosomal assemblies for the Tibetan Antelope [33] and the blind mole rat [34] as well as for the comparison of chromosome evolution among several avian species [35]. RACA can therefore be applied to the issue of fragmentation in the draft red fox genome, as the genomes of two close phylogenetic relatives of the fox have genome assemblies that include chromosomes. The dog (*Canis lupus familiaris*) and red fox shared an ancestor 9 to 10 million years ago [36], and the dog reference genome was sequenced and assembled into chromosomes using Sanger sequencing technology along with the development of tools including a Bacterial Artificial Chromosome (BAC) library and a radiation hybrid map of the dog genome [17]. Likewise, the cat (*Felis catus*) genome has been assembled into chromosomes [20] and offers a natural outgroup to the dog and fox, given that the canid and felid branches of Carnivora diverged about 50–60 million years ago [36]. 

An apparent limitation to using the dog and cat to assemble the fox chromosomes with RACA is the significant chromosomal rearrangement on the canid branch of the carnivore phylogenetic tree. The cat karyotype, which is comprised of 16 bi-armed and 2 single-armed autosomes [37], is thought to be similar to that of the ancestral carnivore [38,39]. While the fox’s karyotype is composed of 16 metacentric autosomes, the sex chromosomes, and 0–8 supernumerary B chromosomes [37], the dog has 38 acrocentric autosomes in addition to its sex chromosomes [37]. Comparison of karyotypes within the carnivore and canid clades suggests that the highly fragmented dog karyotype is very similar to the ancestral canid karyotype, and thus the dog-fox ancestor [40]. Syntenic blocks corresponding to regions of chromosomes conserved between the dog and fox have been characterized with comparative cytogenetic analysis [40,41] and with fluorescence in situ hybridization (FISH) using both flow-sorted dog chromosomes [42,43] and dog-derived BAC clones [44]. Synteny between the two species persists even at moderately high resolution: Construction of the red fox meiotic linkage map using canine microsatellite markers revealed that the order of the markers is almost completely conserved within dog and fox syntenic blocks [45,46,47,48,49]. These studies have demonstrated that each fox autosome is syntenic to at least two dog autosomes, and most dog autosomes map to the fox karyotype in a single syntenic block, although four dog chromosomes map in two blocks [40,42,44,46]. The relationship between the cat, fox, and dog chromosomes has also been established using FISH [50], and synteny between the dog and cat chromosomes has been characterized at a resolution of about 1 Mb using radiation-hybrid (RH) mapping [51]. Due to the lack of a meiotic or comparative RH-map, fox-cat synteny has never been examined at moderate- or high-resolution. The existing interspecies comparisons [50,51] suggest that dog and cat share much smaller syntenic blocks than dog and fox, consistent with the phylogeny. 

The new red fox assembly sits at the intersection of traditional genome assembly pipelines and more modern ones: though it was assembled using the cost-effective short-read Illumina sequencing pipeline, two of its close phylogenetic relatives have Sanger-sequenced genomes that include full chromosome assemblies, and both physical and linkage mapping have established the relationships among the fox and dog chromosomes. The wealth of existing resources positions the new red fox genome assembly to both benefit from and contribute to the known relationships among carnivore and canid genomes. In the present study, the genomic sequence comprising the fox scaffolds was contextualized through sequence-level alignment to the dog reference chromosomes alongside a parallel alignment of the cat genome to the dog genome. RACA was used to combine this comparative genomic information with the raw sequence reads generated during genome assembly [12] to identify stretches of sequence colinear in dog and fox. These RACA fragments were then integrated with the dog-fox comparative chromosome map and the fox meiotic linkage map to assemble RACA’s fragments into large fragments on the scale of fox chromosome arms. 

Addressing and refining the errors introduced by assembly from short sequencing reads will facilitate evolutionary and population genetic analyses in the red fox and the mapping of the genetic architecture of quantitative traits of interest. Capitalizing on the products of early efforts in mammalian comparative genomics, namely the cat and dog reference genomes, to improve the fox genome assembly also advances the resolution at which karyotype evolution among the canids can be elucidated. The scaffolds of the red fox genome thus provide a valuable template for the construction of fox chromosomal fragments that can advance both organismal and comparative analysis of the red fox. 

## 2. Materials and Methods 

Syntenic chains and nets use interspecies alignments to identify runs of synteny between the species. In order to construct the chains and nets, the dog chromosomes comprising CanFam3.1 [17] were first partitioned into 40,010-bp pieces with 10 Kbp of overlap between pieces, and the 500 largest fox scaffolds (50 Kb and larger) (NCBI BioProject 378561) and the cat chromosomes from FelCat5 [5,20] were partitioned into 20-Kbp, nonoverlapping pieces. Fox and cat fragments were aligned against the dog fragments using LASTZ v1.02.00 [52,53]. For the fox-dog alignment, the LASTZ parameters included a gap opening penalty (O) of 600, a gap extension penalty (E) of 150, a minimum score threshold for inclusion of an alignment on the first pass (K) of 4500 and on the second pass (L) of 2200, a minimum score threshold for interpolation (H) of 2000, and the default LASTZ scoring matrix. Chains and nets had previously been developed for alignment of a previous version of the dog genome (CanFam2) with a previous version of the cat genome (FelCat3) [5], so the parameters from that alignment as listed by UCSC (University of California, Santa Cruz, CA, USA) Genome Browser were used in the current analysis. The LASTZ parameters were set to use the default scoring matrix and O = 400, E = 30, K = 3000, L = 2200, H = 2000, and M = 50, with M specifying the threshold at which a sequence is considered to be repetitive and excluded from additional seeding. LASTZ alignments were run in parallel on the Carl R. Woese Institute for Genomic Biology’s (University of Illinois at Urbana-Champaign, IL, USA) high-throughput computing cluster.

The alignments were merged to create chains and then nets, which are ungapped and gapped syntenic fragments (respectively), using the standard algorithms from the kentUtils v302 [54,55]: axtChain, chainSort, chainNet, and netSyntenic. The parameters in the chaining step for dog and fox included a minScore cut-off parameter of 5000 and a linearGap parameter set to medium. For cat and dog, the chaining parameters were set to a score threshold of 3000 and a linearGap of medium. The output of the chaining and netting step was a chain (.chain) and a net (.net) file corresponding to each dog chromosome compared to the fox, and the same for each chromosome compared to the cat. 

The raw Illumina sequencing libraries (Table S1) generated in the original sequencing of the genome (NCBI BioProject 378561) were aligned against the largest 500 scaffolds in the draft genome using Burrows-Wheeler Alignment tool (BWA) 0.7.7 [56]. The alignments were converted into RACA’s input format using the Perl script available on the RACA website [57]. Additionally, insert size statistics were measured for each of the 15 libraries using Picard v. 1.108 (Broad Institute, MA, USA) to calculate the mean and standard deviations of insert size mapping against the fox scaffolds (Table S1). 

RACA (v. 0.9.1.1) [32] takes as input the dog-fox and dog-cat chains and nets, the insert size distributions, and the output of the Perl script from the RACA website. Additionally, RACA requires that the phylogenetic relationships among the three species be provided as a Newick tree, so this tree was estimated using the syntenic nets (Appendix A). The default RACA parameters were used for Window Size, Intracoverage Percentage, and Insert Size Threshold. RACA was run four times in order to test the block resolution parameter at 40, 80, 100, and 150 Kbp to identify its optimal value. 

RACA evaluates the probability that fragments are adjacent based on support from overlapping sequencing reads; however, in the case of fox-dog synteny, strong a priori predictions of adjacency were available from previous analyses of interspecies synteny [40,42,43,44,46]. For this reason, the order of the markers and the known syntenic relationships between the dog and fox chromosomes were considered to be more reliable indicators of fragment order than RACA’s fragment adjacency estimates. The number of dog chromosomes identified as syntenic to each scaffold by RACA was compared to their syntenic relationships with the dog genome as previously predicted [12]. The hypothesis that shorter scaffolds were more likely to be excluded by RACA, given that they would be less likely to contain runs of sequence longer than the Minimum Block Resolution, was tested using a one-tailed Welch Two Sample *t*-test in R [58] to compare the log10-transformed scaffold lengths. 

The 414 microsatellite markers used previously in construction of the fox meiotic linkage map [45,46] were aligned to the fox genome. The primers for each marker were mapped with Bowtie 2 [59] to the fox scaffolds and then to the RACA fragments as though they were reads from paired-end sequencing. The insert size was set to a maximum of 700 and –D (the number of consecutive seed extension attempts that can fail before Bowtie 2 [59] skips ahead) was set to 50. Primer sets that mapped concordantly were considered to map robustly and were assigned a location. 

The meiotic positions of these markers on the fox chromosomes, as well as their physical and genomic positions in the dog, are already known, so identifying them within the fox genome sequence served to bridge the gap between the linkage map and the contents of the genome. The RACA fragments at each of the tested block resolutions were then checked against the meiotic linkage map to identify extent of the concordance between the marker order in the fragments and their known order. The resolution at which the RACA fragments most faithfully recapitulated known marker order was 40 Kbp, so these fragments were used in all subsequent analyses. 

Assembly of the fox chromosomes then proceeded in two steps: first, all RACA fragments mapping to a single dog chromosomes were concatenated based on the order of the microsatellite markers (first) and then according to known dog-fox synteny. When the dog sequence syntenic to two adjacent RACA fragments was not continuous, gaps were inserted based on the missing syntenic sequence in the dog. In the next step, the new fragments were assigned positions along the fox chromosomes based on the known order and direction of the dog chromosomes relative to the fox chromosomes and the order of the markers on the fox chromosomes [42,46]. Unless they were mapped continuously in a single scaffold, segments of fox chromosomes that comprise distinct dog-fox syntenic blocks were assembled into separate fox chromosomal fragments because no assumptions could be made about the amount of sequence separating them. RACA also provided the position(s) in the cat genome corresponding to each RACA fragment, so the order of the fragments in cat was compared to cat-dog synteny as established by radiation hybrid mapping [51] and comparative cytogenetics [50] to confirm that the fragments recapitulated known cat-dog synteny.

Dog chromosomes CFA1, CFA13, CFA18, and CFA19 map in multiple syntenic blocks to the fox genome and correspond to evolutionary breakpoints. The exact breakpoints of the syntenic blocks along the fox chromosome have not previously been identified (Table S2). The RACA fragments syntenic to these four dog chromosomes were examined at high resolution and compared against the previous mapping of the breakpoints [44] to refine the syntenic positions of the breakpoints in the dog genome.

Finally, the fox chromosomal fragments were assembled as FASTA files using a Python script (available at https://github.com/rando2/foxmap) to extract genomic sequence from the scaffolds according to the positional ranges identified by RACA. Directionality of the fragments was determined based on, first, the orientation of the scaffold relative to the rest of the chromosomal fragment, and, second, the likely direction of that fragment in the fox genome based on dog-fox synteny [46]. Sequences from the scaffolds were reversed and complemented using Python as necessary. Gaps of 100 bp were added between scaffolds within a RACA fragment according to RACA’s recommendation. Gaps between RACA fragments within fox chromosomal fragments were determined based on the space between their respective syntenic positions in the dog.

Pairwise alignments using the new fox genome chromosomal assembly as the reference and the dog (CanFam3.1) and cat (FelCat8) genomes as targets were generated with LASTZ v.1.02.00 [52,53] using the following parameters C = 0, E = 30, H = 2000, K = 3000, L = 2200, and O = 400, with C=0 specifying chaining should not be used (--nochain) but gaps can be used (--gapped). The resulting pairwise alignments were converted into the UCSC chains and nets alignment formats with axtChain [54,55] (parameters: −minScore = 1000, –linearGap = medium, and –verbose = 0) followed by chainAntiRepeat, chainSort, chainPreNet, chainNet, and netSyntenic, all with default parameters. Pairwise homologous synteny blocks were defined using the maf2synteny tool [60] at 300-Kbp resolution and uploaded to Evolution Highway [61] (http://eh-demo.ncsa.illinois.edu/).

## 3. Results

### 3.1. Interspecies Synteny

Large-scale parallel alignment of the largest 500 fox scaffolds (94% of the sequence in the draft genome) against the dog genome with LASTZ [52,53] and analysis with the kentUtils [54,55] produced chain and net files that define collinear sequence fragments representing synteny between the fox scaffolds and the dog chromosomes. Comparable alignments were produced of the cat and dog genomes. 

### 3.2. Reference-Assisted Chromosome Assembly

With 40-Kbp block resolution, RACA identified 537 conserved blocks ranging in size from 41.4 Kbp to 54.7 Mbp of fox sequence, with sizes of the blocks ranging from 42.3 Kbp to 53.8 Mbp in the dog. The conserved blocks represent single, continuous regions on both a dog chromosome and a fox scaffold. RACA also provided corresponding location(s) in the cat genome for fragments that were homologous in all three species. Once read alignment information from the original genome sequencing reads was integrated, RACA merged these conserved blocks and added additional blocks to fill in gaps, resulting in a set of 128 RACA fragments. The RACA fragments ranged in size from 34.9 Kbp to 104.6 Mbp. RACA’s assembly closely followed the structure of the individual dog chromosomes, except for one fragment spanning both dog chromosomes 12 and 33 (CFA12 and CFA33), which together comprise the larger arm of fox chromosome 1 (VVU1q) [40]. Mapping an independent set of fox paired-end reads revealed support for 12.2% of the scaffold adjacencies suggested by RACA (Appendix B).

**Figure 1:**
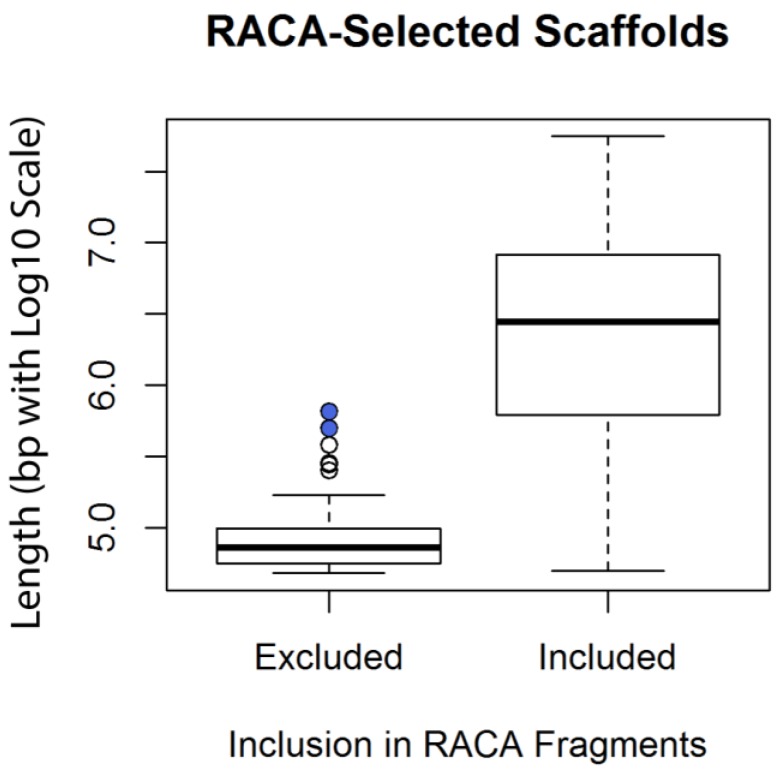
Box plot indicating lengths of scaffolds included and excluded from Reference-Assisted Chromosome Assembly’s (RACA’s) assembled fragments. RACA was provided with the largest 500 scaffolds, which ranged from 48 to 55,683 Kbp. The scaffolds included in the assembly spanned approximately the full range of sizes (50 to 55,683 Kbp) whereas the excluded scaffolds tended to be smaller (48 to 656 Kbp). The outlier points shaded in blue represent scaffolds 292 and 310, which were experimentally demonstrated to contain red fox Y-chromosome sequence [14], and therefore would not be expected to be included in RACA’s assembled fragments.

RACA incorporated sequence from 398 scaffolds into the fragments it assembled, meaning that 102 scaffolds analyzed by RACA were not included in the assembled fragments. Although the scaffolds used by RACA spanned the full range of represented scaffold lengths (50 Kbp to 55 Mbp), the excluded scaffolds were concentrated at the short end of the range (Figure 1). Comparing the log10 of the nucleotide lengths of the 500 scaffolds revealed that the scaffolds excluded from the assembly (mean: 4.91; standard deviation: 0.22) were significantly shorter than those included (mean: 6.31; standard deviation: 0.76) based on a Welch two sample *t*-test (*t* (491.01) = −31.845, *p* < 2.2 × 10^−16^). Six of the excluded scaffolds have been reported [12] as mapping to the dog Y-chromosome, which was not included in the present analysis. 

**Figure 2:**
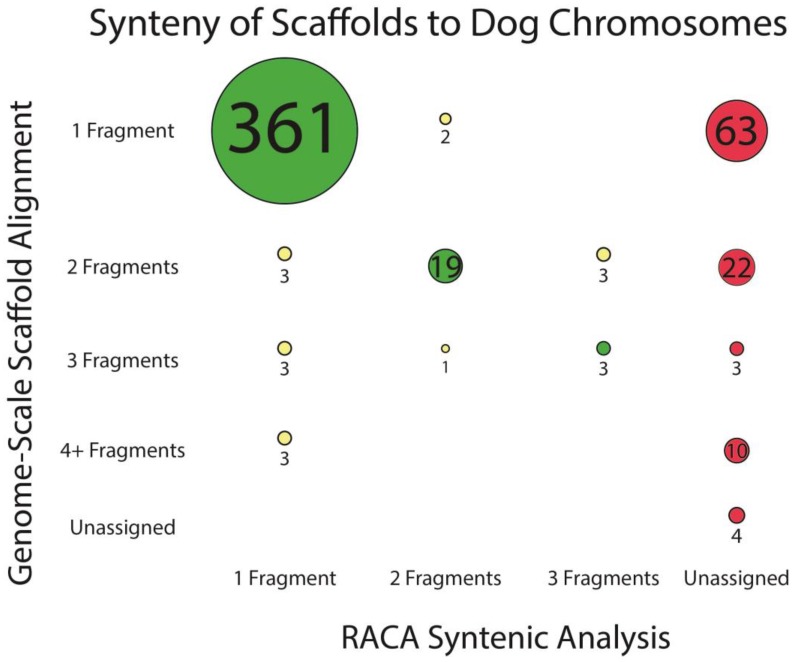
The number of distinct dog chromosomes syntenic to each scaffold, as predicted with two complementary methods: genome-scale alignment to identify syntenic dog chromosome(s) for each scaffold [12], and the number of dog chromosomes syntenic to each scaffold as identified by RACA. Circle diameter is proportional to the number of scaffolds, which is presented inside of or beside the circle. Green indicates that the same number of syntenic chromosomes was predicted by both methods; yellow that the methods predicted different numbers; and red that the scaffolds were excluded from the RACA assembly. Unlike the prior analysis, RACA did not compare the scaffolds to the dog Y-chromosome.

The same prior analysis [12] had identified 70 scaffolds among the largest 500 as putative bioinformatic chimeras, meaning they were likely to contain misassembled sequence drawn from multiple fox chromosomes. Most of the scaffolds predicted to be syntenic to two or more dog chromosomes were split into the predicted number of fragments by RACA (Figure 2).

### 3.3. Assembly of Chromosome-Scale Fragments

The RACA fragments were integrated with the fox meiotic linkage map, which was constructed from dog-derived microsatellite markers whose order is known to be conserved along the fox chromosomal arms [46]. The microsatellite markers were mapped in silico onto the scaffolds and the RACA fragments to determine the order of the RACA fragments along the fox autosomes and X-chromosome. Of the 414 markers available, Bowtie 2 [59] mapped 373 concordantly to the largest 500 fox scaffolds, and all of those fell within RACA fragments (Table S3). Seventy-two of the 128 RACA fragments contained at least one marker, and, of those, 57 contained two or more markers. The largest number of markers in a single RACA fragment was 25. 

Presence of one or more markers allowed for the position of each fragment relative to the other fragments to be determined according to the fox meiotic linkage map, and when two or more markers mapped to a fragment, its direction relative to the other fragments could also be inferred. Fifty-six RACA fragments did not contain markers and were assigned positions along the fox chromosome arms according to their syntenic positions on the dog chromosomes and the previously identified patterns of fox-dog synteny (i.e., in the same order and orientation they would fall within the dog genome). Gaps were added between RACA fragments to be consistent with the corresponding unassembled dog sequence. Scaffolds 1 and 9, which are likely to overlap a historical fusion event, were recombined at the site where RACA had recommended splitting them because the order of the markers within each scaffold indicated that each scaffold contained sequence corresponding to two adjacent dog-fox syntenic blocks. Through this procedure, the number of fragments was reduced to 40. These chromosome-scale fragments ranged in size from 20.2 to 124.0 Mbp. All chromosomal fragments contain markers placed on prior linkage maps (Table S3).

Comparison of the order of the 358 markers in RACA fragments that contained two or more markers (Table S3) to previous estimations of marker order [46,47,48,49] revealed a high level of congruence between the marker order in the fragments assembled by RACA, and as estimated with linkage. In all but 10 cases, the order of the markers in the assembly matched at least one previous map. 

### 3.4. Refinement of Fox-Dog Synteny at Chromosomal Breakpoints

All but four dog chromosomes correspond to single syntenic blocks in the fox genome. CFA 1, 13, 18, and 19 each split into two distinct syntenic blocks when compared to the fox chromosomes, and previous studies have sought to characterize the dog genomic positions of the breakpoints in dog-fox synteny (Table 1). The RACA fragments homologous to these four dog chromosomes were analyzed to determine the syntenic position of the each relative to the fox breakpoint. Several RACA fragments were anchored in regions with known syntenic placement on the fox map but extended into the regions of the dog chromosomes whose locations relative to the breakpoint was unknown. These RACA fragments allowed for the size of the region unmapped to either the syntenic block to be reduced and refined the dog genomic positions of the breakpoints in fox (Table 1). One RACA fragment, 1a, contains sequence that is syntenic to either side of the breakpoint on dog chromosome 1 (209,340–423,404 bp and 24,994,866–25,534,824 bp); a lack of markers in this fragment prevented confident assignment to either fox chromosome 1 or 5, but it has been assembled with RACA fragment 1b to form fox chromosomal fragment VVU5p-proximal, which is the segment of fox chromosome 5 located proximally (that is, adjacent to the centromere) on the p arm.

### 3.5. Assembled Chromosomes

**Figure 3:**
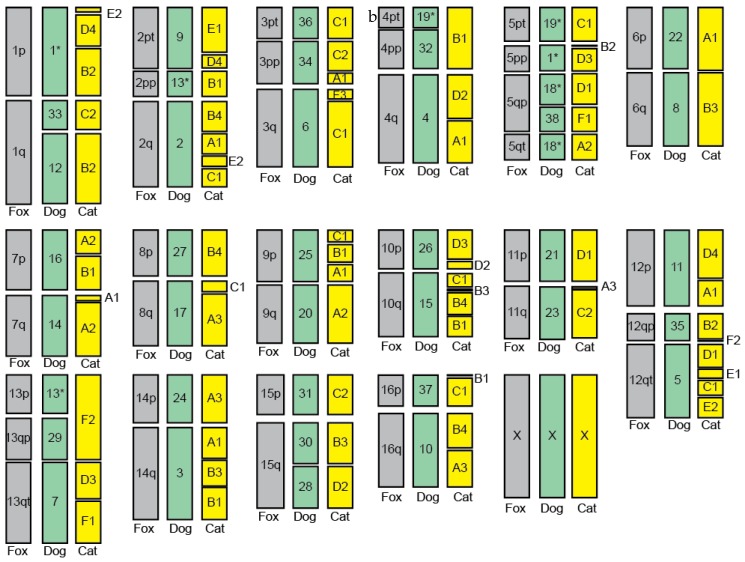
The fox chromosomal segments shown alongside the corresponding syntenic chromosomes in dog and cat. Fox chromosomal fragments are ordered to approximate the full assembled chromosome, and chromosome numbers appear inside of or alongside fragments. Asterisks indicate dog chromosomes that map in more than one syntenic block to fox. Dog and fox fragments are to scale; cat fragments are approximately to scale. Syntenic blocks smaller than 500 Kbp (found only for the cat) are not shown.

The sequences of the 40 assembled chromosomes are available online as NCBI BioProject 421766 and represent version 2.4 of the red fox genome assembly (vv2.4). Comparing the order of the RACA fragments across the three assemblies allowed for an interspecies comparative chromosome map to be inferred (Figure 3). Synteny between the chromosomal fragments and the dog and cat chromosomes was consistent with previously characterized synteny among the three species [40,42,43,44,50] with one exception: The cat chromosomes found to be syntenic to the regions of VVU1 and VVU5 that are syntenic to CFA1 were swapped relative to the previous dog-fox-cat comparative karyotype [50], but the positions in the comparative karyotype proposed here were consistent with the dog genomic positions syntenic to each cat chromosome as characterized with RH-mapping [51] and the known fox breakpoints [44]. 

**Figure 4:**
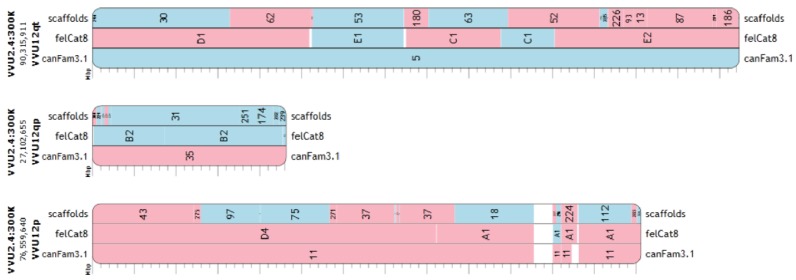
View of fox, dog, and cat synteny along fox chromosome 12. Interspecies chromosome-scale synteny has been visualized in Evolution Highway [61]. The fox scaffolds comprising vv2.2 and the final 40 fragments comprising vv2.4 are included in the visualization. Red represents fragments that run negative relative to the fox chromosome fragments (i.e., opposite strand), whereas blue fragments run in the same direction.

Additionally, the high-resolution analysis identified previously unknown, short (between 20 Kbp and 11.9 Mbp) cat-fox syntenic blocks, many of which are consistent with dog-cat synteny as characterized in the dog-cat comparative RH map. The cat-fox comparison in Figure 3 is inferred based on the two species’ alignment to the dog. As previously reported [50], synteny is conserved between fox and cat but not between fox and dog on VVU4 and VVU13 (Table 2). The high-resolution analysis revealed three cases (VVU1, VVU6, and VVU7) where fox sequence syntenic to a continuous region in the cat genome was split across both arms of a single fox chromosome (Table S4). Synteny among fox, dog, and cat, with positions from both the fox draft genome and the chromosome assembly, are visualized in Evolution Highway [61] (Figure 4).

In almost all cases, the assembled fox chromosomal fragments split at locations where synteny breaks with the dog, such that the fragment is syntenic to a single dog chromosome. Three exceptions were found. Fox chromosomal fragment 1q was assembled by RACA based on read support along scaffold 7 to allow the fusion of regions syntenic to dog chromosomes 33 and 12. Additionally, fragments VVU5qp (the proximal segment of the larger (q) arm of fox chromosome 5) and 15q are cases where a scaffold bridges two adjacent dog-fox syntenic blocks. Although RACA did not suggest leaving the scaffold intact, the marker order indicated that the scaffold was likely to overlap an historical fox fusion. Thus, there are 40 fox chromosomal fragments in the current assembly, corresponding to 43 syntenic blocks between fox and dog. A full comparison of synteny among the three species across all fragments is provided in Table S4. 

## 4. Discussion

The Genome 10K Project seeks to propel the coming-of-age of comparative genomics by sequencing a phylogenetically comprehensive set of vertebrate genomes. This objective was advanced, at least in the case of the red fox genome, by earlier research in comparative genomics that provides the resources needed to overcome the computational limitations of genome assembly with short-read NGS technology. Here, the scaffolds comprising the red fox genome were examined through the lens of comparative genomics to construct 40 fragments corresponding to partial or full fox chromosome arms. This pipeline allowed for the resolution of some assembly errors (e.g., chimerism) and for the red fox genome sequence to be reconciled with the red fox meiotic linkage map. The assembly of the red fox draft genome into chromosome-scale fragments will facilitate future genomic analysis of the fox and is thus a necessary step in the development of this genome. 

The red fox genome project is positioned to build on resources developed over more than two decades that include the Sanger sequencing and OLC assembly of the chromosomes of two phylogenetically close species [5,17,20], characterization of the red fox chromosomes relative to the dog chromosomes through comparative cytogenetic analysis and FISH [40,41,42,43,44], and the development and refinement of a red fox meiotic linkage map using dog-derived markers [46,47,48,49]. RACA identified synteny between the red fox scaffolds and the dog chromosomes, and the placement of the markers along RACA’s fragments allowed for the assembly of 40 fragments ranging in size from 20.2 to 124.0 Mbp. The assembled fragments comprise 2.38 Gbp of sequence including 2.34 Gbp of scaffold-derived sequence (i.e., excluding the gaps added by RACA or to combine RACA fragments). The chromosomal fragments contain 93.7% of the 2.5 Gbp of sequence originally reported [12]. Additionally, a minimum of 0.05% of the sequence contained in the original genome is derived from the Y-chromosome [14]. Thus, although the refined fox assembly incorporates genomic information from only 398 large scaffolds of the 676,878 scaffolds assembled, this corresponds to a loss of less than 3.5% of sequence information from the draft genome. 

The fox chromosomal fragments are highly consistent with previous work using meiotic linkage mapping, with the order of the markers in the RACA fragments being almost identical to the established marker order on the fox map (Table S3). This concordance allowed for marker-guided assembly of the RACA fragments into the full and partial chromosomal arms. The ten discrepancies in marker order along the RACA fragments were placed in or near regions known to be affected by high levels of pericentromeric suppression in the fox [46] (Table S3). The placement of the markers in the RACA fragments therefore not only facilitated the assembly of the chromosomal fragments, but also offered new insight into regions that have been difficult to resolve using meiotic linkage. 

Furthermore, explicitly developing the fox as a resource can provide additional support for research in chromosome evolution. The Canidae and Felidae branches of Carnivora diverged approximately 60 million years ago, and within the canids, four major clades are recognized: red-fox-like canids, South American canids, wolf-like canids, and the basal *Urocyon* clade [17]. The dog is a wolf-like canid, and the red fox belongs to its eponymous clade. While comparison of chromosomal rearrangements between the fox, dog, and cat clearly support the closer phylogenetic relationship between the dog and fox, there are at least two regions where synteny between the cat and fox chromosomes is more highly conserved than between dog and fox. The regions of VVU4 syntenic to cat chromosome B1 and of VVU13 syntenic to cat chromosome F2 have been found to form continuous syntenic blocks in cat [50] and canid lineages other than the wolf-like canid clade [44]. The present high-resolution analysis revealed regions where the evolutionary history may be more complex than previously known (Table S4). For example, on VVU6, RACA identified the regions corresponding to the telomeres of CFA22 and CFA8 (e.g., the VVU6 centromere) as being syntenic to cat chromosome A1. However, it does not appear that this region forms a continuous syntenic block with cat because there is 6.5 Mbp of missing cat sequence that RACA identified as being syntenic to VVU7 and these regions are not adjacent in other canids [39,44]. Similarly, sequences syntenic to cat chromosome B2: 31.9–154.2 Mbp split into multiple syntenic blocks across VVU1p and VVU1q, whereas these regions are syntenic to distinct chromosomes in the dog (CFA1 and CFA12, respectively). A similar pattern is found for the regions of VVU7p and VVU7q that are syntenic to cat chromosome A2. Whether or not these breakpoints have been reused [62,63,64] is a question for future analysis. Regardless, the strong support for at least two derived fissions in the wolf-like canids as identified by both physical [44,50] and now sequence-level synteny suggests that two modifications to the estimated ancestral canid karyotype [39] are needed. These modifications would reduce the number of unknown fragments in the ancestral canid karyotype (Appendix C).

High-resolution mapping of dog-fox syntenic blocks can elucidate regions associated with chromosome evolution. Twenty-six fusions and 4 fissions separate the red fox karyotype from the dog karyotype [42], with most fox chromosome arms corresponding to one or more acrocentric dog chromosomes. Identifying the sequence of the fox chromosomes presents an opportunity to characterize the regions associated with these fissions and fusions, especially in the cases where a single scaffold spans a historical fusion in the fox lineage (VVU1q, VVU5qp, and VVU15q). Likewise, analyzing the genomic content of the regions surrounding the four fissions can provide insights into chromosomal evolution in Canidae and beyond. The four fissions correspond to the breaks in dog-fox synteny seen on dog chromosomes 1, 13, 18, and 19. These loci are considered evolutionary breakpoint regions (EBRs) [44], which are positions along the chromosomes known to be the sites of a large number of fission events [39,40,44,50,65,66]. Based on the syntenic patterns observed among canid species [43,44,50], the breakpoints in the fox associated with CFA1, CFA13, and CFA19 all emerged from the same pattern of chromosome evolution: They likely belonged to distinct chromosomal fragments in the ancestral canid that fused in the ancestor of modern wolf-like and South American canids, so they remain separate fragments in the rest of the canids, including the red fox. However, the evolutionary history of the breakpoint on CFA18 is more complex and suggests reuse of this EBR within recent canid chromosome evolution. Previous analysis [44] identified two syntenic blocks corresponding to CFA18 in both the red fox and the grey fox (*Urocyon cinereoargenteus*), but these regions form a single syntenic block in not only the wolf-like and South American canids but also some of the red-fox-like canids (i.e., raccoon dog and beat-eared fox). Becker and colleagues [44] mapped the breakpoints in the red fox and grey fox to the same regions along CFA18 but concluded that two distinct fusion events would best explain the pattern observed. They also noted that this syntenic block is adjacent to the CFA38-syntenic block not only in the red fox, but also in the grey fox, which belongs to the mode basal canid clade; this pattern is consistent with two separate events where these syntenic blocks were fused in the past 10 million years of canid evolution [44]. On VVU5qp in the proposed assembly of the fox chromosomal fragments, we present the sequence composition of an interstitial boundary of syntenic blocks corresponding to CFA38 and CFA18, although CFA38 and CFA18 share two boundaries on VVU5q, and the boundary assembled in scaffold9 is not the one recapitulated in the *Urocyon* karyotype. While the sequence of this EBR would be of particular interest to future analysis, the red fox chromosomal assembly, as a whole, will allow for higher resolution characterization of EBRs and the genomic content underlying chromosome evolution. 

The chromosome fragments assembled here are essential for the advancement of genomic studies in the fox. Previous studies seeking to map behavioral traits in the fox have been forced to choose between relying on low-resolution microsatellite markers (e.g., [49]), interpreting against the dog genome assembly (e.g., [67]), or analyzing short genomic fox fragments (e.g., [12]). The large fragments will facilitate the use of high-resolution markers and the use of the fox as a reference genome for sequence alignment, while still allowing continuous analysis along fragments on the scale of chromosomal arms. The scale of the new fragments will facilitate the mapping of phenotypic traits, including the extreme behavioral phenotypes of the Novosibirsk farmed populations that influenced the inclusion of the red fox in the Genome 10K Project, and will advance future studies using in evolutionary and population genomics. Additionally, comparative analysis of wild fox populations and farmed foxes selected for behavior or other traits will be facilitated by the longer, continuous runs of sequence. For example, selective sweeps that reduce heterozygosity in regions surrounding a locus of interest will be more easily detected and the correct loci targeted when the sweep is recognized as contiguous, rather than being split across multiple scaffolds. Thus, the fragments themselves provide a new resource for analysis in the red fox. 

Though reorganizing the scaffolds as chromosomes is important to the usability of the red fox genome, this assembly remains unfinished. As sequencing technologies and pipelines continue to evolve, new approaches to resolution of repetitive regions are becoming increasingly feasible. With the new technologies sometimes referred to as third-generation sequencing (3GS), sequencing reads of up to 20 Kbp are now available. The major limitation to many 3GS technologies is a high error rate compared to Sanger or short-read sequencing technologies [68]. Integrating reads from long-read platforms such as PacBio or Oxford Nanopore with short-read Illumina sequencing that can be used to correct errors has proven a valuable strategy for resolving complex regions of genomes [69,70]. In fact, new assembly algorithms have been developed to leverage both short- and long-read NGS technologies. One example is DBG2OLC, where short reads are assembled with de Bruijn graphs into contigs that are then used to correct errors in long reads, allowing for use of an OLC approach [68]. Approaches to sequencing itself have also emerged that obtain spatial information and sequence information simultaneously. These include Hi-C, which elucidates chromatin structure [71], and optical mapping, where fingerprints indicating the positions of restriction enzymes are visualized along long stretches of the genome [72]. Selection of individuals for sequencing can also play a role in improving de novo assemblies through strategies such as trio-binning, where sequencing parents alongside offspring facilitates the identification of specific haplotypes in a departure from the conventional choice to sequence inbred individuals [73]. Short-read-assembled genomes like that of the red fox will benefit significantly from the improvements available with these approaches as 3GS becomes more widely available and economical.

Beyond the red fox itself, the chromosome fragments here and future improvements to them will offer a resource for analyses of chromosome evolution within Canidae. The scaffolds that contain sequence from two adjacent syntenic blocks offer an opportunity to analyze the genomic sequence comprising breakpoints during recent canid karyotype evolution, and, in one case an EBR, because the red fox is the first canid other than dog for which a genome has been assembled. Therefore, just as the red fox assembly has benefited greatly from the work of comparative mammalian genomics, it can also offer new insights into karyotype evolution in Canidae, the family with one of the most highly rearranged karyotypes [63]. The assembly of the fox draft genome into large fragments is thus a critical next step for genomic research in the red fox itself and in the red fox genome’s potential to contribute to comparative mammalian genomics.

## Figures and Tables

**Table 1:** Refinement of gaps in synteny between dog and fox. Four dog chromosomes map in more than one syntenic block to the fox genome, meaning a single dog chromosome is syntenic to more than one contiguous region of the fox as the result of either a fission event in the dog lineage or a fusion event in the fox lineage. The breakpoints and sizes of the unassigned regions as refined by previous analysis [44] are indicated in the third and fourth columns, respectively. High-resolution mapping in the present study allowed for refinement of the breakpoints, as indicated in the fifth and sixth columns. All genomic locations are based on CanFam3.1. RACA fragment 1a is excluded due to its lack of markers.

Fox Chromosomes	Dog Chromosome	Previously Unassigned Positions on Dog Chromosome (bp)	Previous Gap Size (Kbp)	Dog Chromosome Positions Still Unassigned (bp)	Updated Gap Size (Kbp)
1 & 5	1	24,600,000–25,700,000	1100	24,988,836–25,579,247	590.4
2 & 13	13	37,800,000–38,600,000	800	38,258,211–38,277,954	19.7
5 (2 fragments)	18	24,400,000–26,000,000	1600	25,259,332–25,332,083	72.8
4 & 5	19	18,800,000–22,100,000	3300	19,878,341–20,333,685	455.3

**Table 2:** Conserved synteny between cat and fox relative to dog. Alignment of the dog genome assembly (CanFam3.1) and cat genome assembly (FelCat5). Top: against VVU4; bottom: against VVU13. In these three cases, fox and cat map in a single syntenic block whereas fox and dog map in two blocks, as indicated by bold text. The cat syntenic blocks spanning two dog syntenic blocks are shaded. Genomic positions in CanFam3.1 and FelCat5 are indicated. * Indicates chromosome comprised by two dog-fox syntenic blocks.

Fox Fragment	Dog Chromosome	Dog Positions (Mbp)	Direction	Cat Chromosome	Cat Position (Mbp)
4pt	19 *	0–19.9	Forward	**B1**	**84.0**–**144.6**
4pp	32	0–38.7	Reverse		
4q	4	0–88.3	Forward	D2	8.6–48.2
				A1	172.6–227.4
13p	13 *	0–38.3	Reverse	**F2**	**0.6–82.8**
13qp	29	0–41.7	Reverse		
13qt	7	0–80.9	Reverse	D3	33.3–71.5
				F1	0.4–41.3; 65.7–68.7

**Figure A1:**
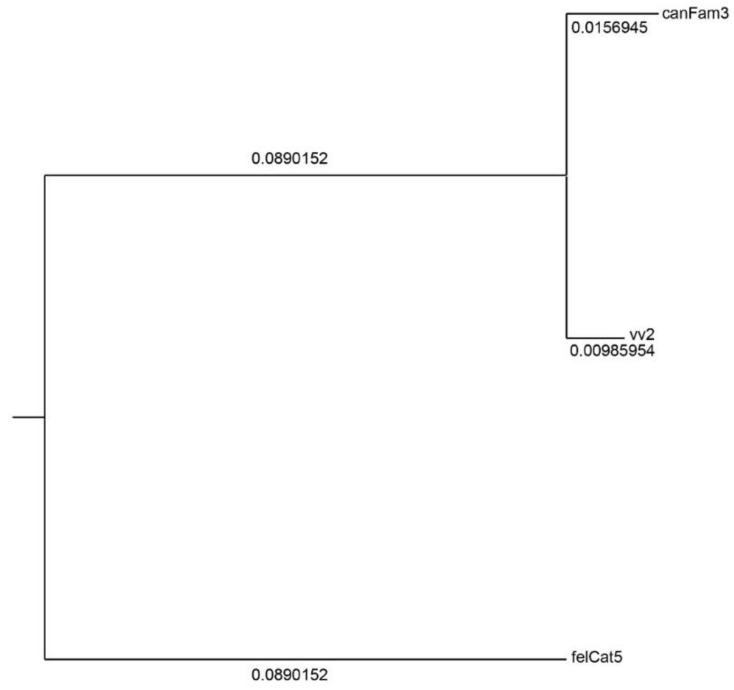
Newick tree visualization including branch lengths.

**Figure A2:**
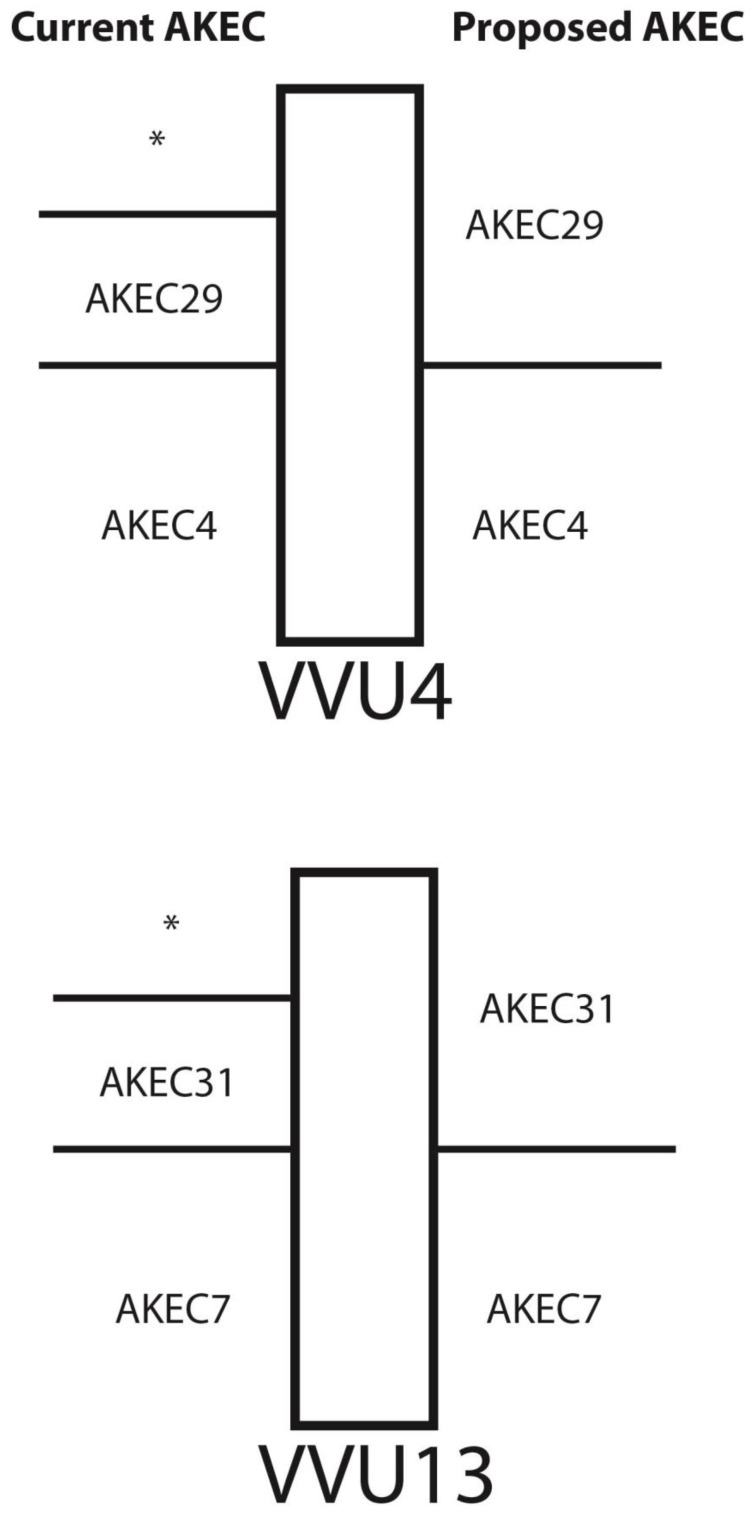
Proposed updates to the Ancestral Karyotype of Extant Canids (AKEC) [39]. Asterisks denote regions of the fox chromosomes where the ancestral chromosome number was unknown in [39]. The original AKEC underestimates the coverage of VVU4 and VVU13 by the previously identified homologous ancestral chromosomes. The results of comparative genome mapping support a simpler homology between the ancestral chromosomes and the modern vulpine chromosomes, as indicated to the right.

## Supplementary Materials

The following are available online at http://www.mdpi.com/2073-4425/9/6/308/s1, Table S1: Libraries used in assembly of the fox genome, Table S2: Assignment of fragments spanning the fox evolutionary breakpoints with dog, Table S3: Marker positions, Table S4: Positions of the fox chromosome fragments in the dog and cat genomes.

## Appendix A

A Newick tree indicating the branch lengths between the target, reference, and outgroup species must be provided to RACA as input. In order to estimate the phylogenetic relationship between the target (fox), reference (dog), and outgroup (cat) species’ genomes, the .net files generated during the creation of the chain and net files were converted to .maf format using netToAxt and axtToMaf from the KentUtils [54,55], then concatenated, and finally analyzed using phyloFit [74]. Within phyloFit, nucleotide substitution rates were estimated under the reversible nucleotide substitution model [75], as suggested in a previous work [76]. The following Newick Tree was produced: ((canFam3:0.0156945,vv2:0.00985954):0.0890152,felCat5:0.0890152)

The Newick tree itself is used by RACA, but Polydendron [77] offers a way to visualize the Newick tree for easier interpretation (Figure A1).

## Appendix B

The goal of the assembly of the fox chromosome fragments was to reorganize the sequence contained in the scaffolds of the draft genome to more accurately recapitulate the structure of the red fox genome. To assess whether the chromosomal fragments represent an improved organization of the fox genome’s sequence content, we analyzed whether some of the scaffold junctions suggested by RACA represented adjacent blocks of sequence. Breaks in the assembly corresponding to different scaffolds would be expected to occur in regions that are difficult to assemble, such as those rich with repetitive elements. Sequencing reads overlapping these boundaries may therefore be relatively rare, but would offer support for RACA’s ordering of the fox scaffold sequences. To identify reads overlapping the RACA scaffold junctions, we analyzed pooled sequencing data from 10 red foxes (NCBI BioProject PRJNA376561; [12]) mapped to both the chromosome fragment assembly (vv2.4) and to the set of 398 scaffolds from vv2.2 that RACA used to assemble the RACA fragments.

The reads were first filtered to remove adaptor contamination and then aligned to both assemblies using Bowtie 2 [59]. For the 360 adjacencies where RACA added a 100-bp gap between the scaffolds, reads were counted if they spanned the gap (i.e., one end on either side of the gap), were oriented with both ends reading towards the gap, and aligned no more than 2000 bp apart based on the insert size reported in the SAM file. These reads were identified using SAMtools version 1.7 [78] to select the concordantly mapped reads that were placed within 5000 bp upstream or 5000 bp downstream from the gaps. The resulting SAM files were analyzed with Python 2.7 (Python Software Foundation, Beaverton, OR, USA) to count cases where one read in a pair was upstream of a gap and the other downstream with no more than 2000 bp between paired ends.

The mapping of the sequencing reads to the chromosome fragment assembly and to the 398 scaffolds used in the RACA assembly produced almost identical results. In both cases, the overall alignment rate was 94.06% and the percent of the reads that mapped concordantly exactly one time was 84.70%. The exact number of reads mapping concordantly exactly one time was slightly different: 345,168,075 pairs mapped to the chromosome fragment assembly and 345,170,193 pairs mapped to the scaffolds. This is a very small difference of 2,118 pairs of reads, or 0.0005% of all reads. This difference is likely due to RACA removing the ends of some of the scaffolds that were used in the chromosome fragment assembly that remained present in the set of scaffolds. In total, 44 of 360 (12.2%) of scaffold adjacencies were covered by sequencing reads.

## Appendix C

The evolutionary origin of modern canid species has been a popular question even since the time of Darwin, and many approaches have been used to address it. One avenue of research has focused on evolutionary analysis of the modern canine/lupine karyotype, which has a large number of chromosomes (2n = 78). Using, at first, interspecies comparisons of chromosome banding patterns [79,80] and, later, comparative cytogenetic analysis with FISH [38,39], studies have sought to reconstruct karyotypes for extinct ancestral species within Carnivora (Ancestral Carnivore Karyotype, or ACK) and Canidae (Ancestral Karyotype of Extant Canids, or AKEC). The proposed ACK is very similar to that of the modern cat, with 2n = 42 [38,39], while the AKEC was estimated to have approximately 2n = 82, with substantial similarity to the modern dog karyotype [38,39]. At the time that the AKEC was published, however, the syntenic positions of several dog chromosomes were unknown. Advances in genomics have allowed for these regions to be mapped in high resolution, and therefore the present study proposes two updates to the AKEC from 2001.

Specifically, two regions were identified where the arrangement of the syntenic blocks in the ancestral canid was likely to have been more similar to the modern cat karyotype than to that of the modern dog. Therefore, the present results, in addition to evidence of these same patterns in other canid lineages including the basal *Urocyon* clade [44], suggest two modifications should be made to the AKEC (Figure A2). The extended homology of AKEC29 and VVU4 would encompass a larger region of Nash’s ACK2q [39], which is homologous to cat B1q. The p arm of VVU4 is not the only region in which complexity would be reduced: based on the results of a comparison across multiple canid lineages [44], AKEC29 would also span Arctic Fox chromosome 6p and Raccoon Dog 5p in their entireties. In the second case, AKEC31 is homologous to cat chromosome F2 and would also be homologous to the full Arctic Fox chromosome 9 and a large interstitial region of Raccoon Dog 1, as depicted previously [39]. Though small changes, these updates reduce the complexity of the relationship of the AKEC and extant canid species and reduce the similarity between the AKEC and the modern dog in favor of species whose genomes have historically been less well characterized.
